# Clinical isolates of uncomplicated falciparum malaria from high and low malaria transmission areas show distinct *pfcrt* and *pfmdr1* polymorphisms in western Ethiopia

**DOI:** 10.1186/s12936-023-04602-6

**Published:** 2023-06-03

**Authors:** Geletta Tadele, Aminata Jawara, Mary Oboh, Eniyou Oriero, Sisay Dugassa, Alfred Amambua-Ngwa, Lemu Golassa

**Affiliations:** 1 Aklilu Lemma Institute of Pathobiology, Addis Ababa University, Addis Ababa, Ethiopia; 2 Medical Research Council Unit the Gambia, London School of Hygiene and Tropical Medicine, Banjul, Gambia

**Keywords:** *Pfcrt*, *Pfmdr1*, Polymorphisms in a gradient of malaria transmission

## Abstract

**Background:**

*Pfcrt* gene has been associated with chloroquine resistance and the *pfmdr1* gene can alter malaria parasite susceptibility to lumefantrine, mefloquine, and chloroquine. In the absence of chloroquine (CQ) and extensive use of artemether–lumefantrine (AL) from 2004 to 2020 to treat uncomplicated falciparum malaria, *pfcrt* haplotype, and *pfmdr1* single nucleotide polymorphisms (SNPs) were determined in two sites of West Ethiopia with a gradient of malaria transmission.

**Methods:**

230 microscopically confirmed *P. falciparum* isolates were collected from Assosa (high transmission area) and Gida Ayana (low transmission area) sites, of which 225 of them tested positive by PCR. High-Resolution Melting Assay (HRM) was used to determine the prevalence of *pfcrt* haplotypes and *pfmdr1* SNPs. Furthermore, the *pfmdr1* gene copy number (CNV) was determined using real-time PCR. A *P*-value of less or equal to 0.05 was considered significant.

**Results:**

Of the 225 samples, 95.5%, 94.4%, 86.7%, 91.1%, and 94.2% were successfully genotyped with HRM for *pfcrt* haplotype, *pfmdr1*-86, *pfmdr1*-184, *pfmdr1*-1042 and *pfmdr1*-1246, respectively. The mutant *pfcrt* haplotypes were detected among 33.5% (52/155) and 80% (48/60) of isolates collected from the Assosa and Gida Ayana sites, respectively. *Plasmodium falciparum* with chloroquine-resistant haplotypes was more prevalent in the Gida Ayana area compared with the Assosa area (COR = 8.4, *P* = 0.00). *Pfmdr1*-N86Y wild type and 184F mutations were found in 79.8% (166/208) and 73.4% (146/199) samples, respectively. No single mutation was observed at the *pfmdr1-*1042 locus; however, 89.6% (190/212) of parasites in West Ethiopia carry the wild-type D1246Y variants. Eight *pfmdr1* haplotypes at codons N86Y–Y184F–D1246Y were identified with the dominant NFD 61% (122/200). There was no difference in the distribution of *pfmdr1* SNPs, haplotypes, and CNV between the two study sites (*P* > 0.05).

**Conclusion:**

*Plasmodium falciparum* with the *pfcrt* wild-type haplotype was prevalent in high malaria transmission site than in low transmission area. The NFD haplotype was the predominant haplotype of the N86Y–Y184F–D1246Y. A continuous investigation is needed to closely monitor the changes in the *pfmdr1* SNPs, which are associated with the selection of parasite populations by ACT.

## Background

*Plasmodium falciparum* resistance to chloroquine, sulfadoxine–pyrimethamine; artemisinin and its partner drugs threatens effective malaria treatment [1, 2]. Worryingly, studies in Africa have indicated the emergence of artemisinin-resistant parasites in eastern region of the continent [3, 4]. Mutations in the *P. falciparum* chloroquine-resistant transporter (*Pfcrt*) gene, specifically, K76T including mutations in three other amino acids in the adjoining K76 (residues 72, 74 and 75), region are considered to contribute to chloroquine and amodiaquine resistance [5]. After CQ withdrawal, studies have shown that chloroquine resistance isolates have gradually reduced in frequency with the circulation of chloroquine sensitive strains [6] as has been reported from different parts of Africa, 84.1% of the parasites became the wild type in Ethiopia [7], 76.3% of isolates were chloroquine sensitive in Kenya [8] and another study in Kenya also showed 93.3% of the parasites were the wild type in 2018 [9], the wild-type *Pfcrt* haplotype CVMNK was found in 22 of the 26 isolates in Niger [10], all detected parasites were the wild type in Malawi in 2001 [11], and a significant reduction in *Pfcrt* 76T from 97.0 to 66.9% in Cameroon [12].

Most previous studies in Ethiopia have shown the fixation of *pfcrt* 76T mutation despite withdrawal of CQ for treatment of uncomplicated falciparum malaria [13–15]. In Ethiopia, the low rate of reversion in CQ-sensitive parasites is associated with the continued use of CQ to treat vivax malaria [16]. Knowledge on the re-emergence of CQ-sensitive parasites would be essential towards the likely reuse of the safe and cheap CQ [17].

*Plasmodium falciparum* multidrug resistance 1 (*pfmdr1*) plays a central role in parasite resistance to artemisinin-based combination therapy (ACT) partner drugs, such as mefloquine, lumefantrine and amodiaquine through single nucleotide polymorphismsand/or gene copy number variations [18, 19]. Thus, protection of the partner drug efficacy is crucial for maintaining the effectiveness of ACT [20]. Mutations at positions N86Y, Y184F, S1034, N1042D, and D1246Y of *Pfmdr1* are suggested to be involved in altered drug transport from the parasite’s cytosol into the digestive vacuole [21]. N86Y mutation increases parasite susceptibility to the partner drugs lumefantrine and mefloquine and the active artemisinin metabolite dihydroartemisinin and conversely augments resistance to the ACT partner drug amodiaquine and the former first-line agent CQ [19]. Studies from malaria endemic sites have reported a significant increase in the wild-type *pfmdr1* N86 and D1246 following ACT [8, 22]. The *pfmdr1,* NFD haplotype (codons N86, 184F and D1246), has been associated with reduced sensitivity to lumefantrine [23] and a temporal increase in the *Pfmdr1* NFD haplotype was reported from different parts of Africa [24, 25].

The dominance of the wide type *pfmdr*-1 N86 and the mutant 184F alleles have been reported from different parts of Ethiopia; all the parasites carried the wild-type *Pfmdr1* N86 [15], 77.3–100% of isolates collected from different sites of Ethiopia carried the *Pfmdr1-*N86 allele (16), and 98.8% and 100% of the parasites carried the wild type *Pfmdr1* N86 and the mutant 184F, respectively [26]. Similarly, a study in South East Ethiopia indicated all the isolates had the wild-type *pfmdr*-1 D1246 [7].

*Pfmdr1* copy number variation (CNV) has been associated with differential susceptibility to anti-malarial, with multiple copies suggesting reduced sensitivity to lumefantrine and mefloquine [27, 28]. In Cambodia, for instance, *pfmdr1* CNV was associated with ACT treatment failure [29]. A study conducted in Kenya showed that post-ACT, parasites had significantly higher *pfmdr1* CNV compared to pre-ACTs [30]. In addition, a study from Sudan showed that *pfmdr1*gene amplification before ACT was associated with recurrent infection during follow-up [31]. Studies have shown different proportion of multiple *pfmdr1* copies, 5.5% in Mynamar [32], 11% in Suriname [33], 20% in Venezuela [34] and 54.2% in Ethiopia [35].

Measuring the prevalence of *pfmdr1* polymorphisms has implications for designing policies such as drug cycling, sequential artemisinin-based combination treatments or multiple first-line therapies [22]. Moreover, monitoring of drug resistance in *Plasmodium* populations is crucial for malaria control and elimination [2]. This study was initiated to determine mutations in *pfcrt* and *pfmdr*1 genes among the clinical isolates of uncomplicated falciparum malaria patients in western Ethiopia's with gradient of malaria transmission.

## Methods

### Study site and period

**Fig. 1:**
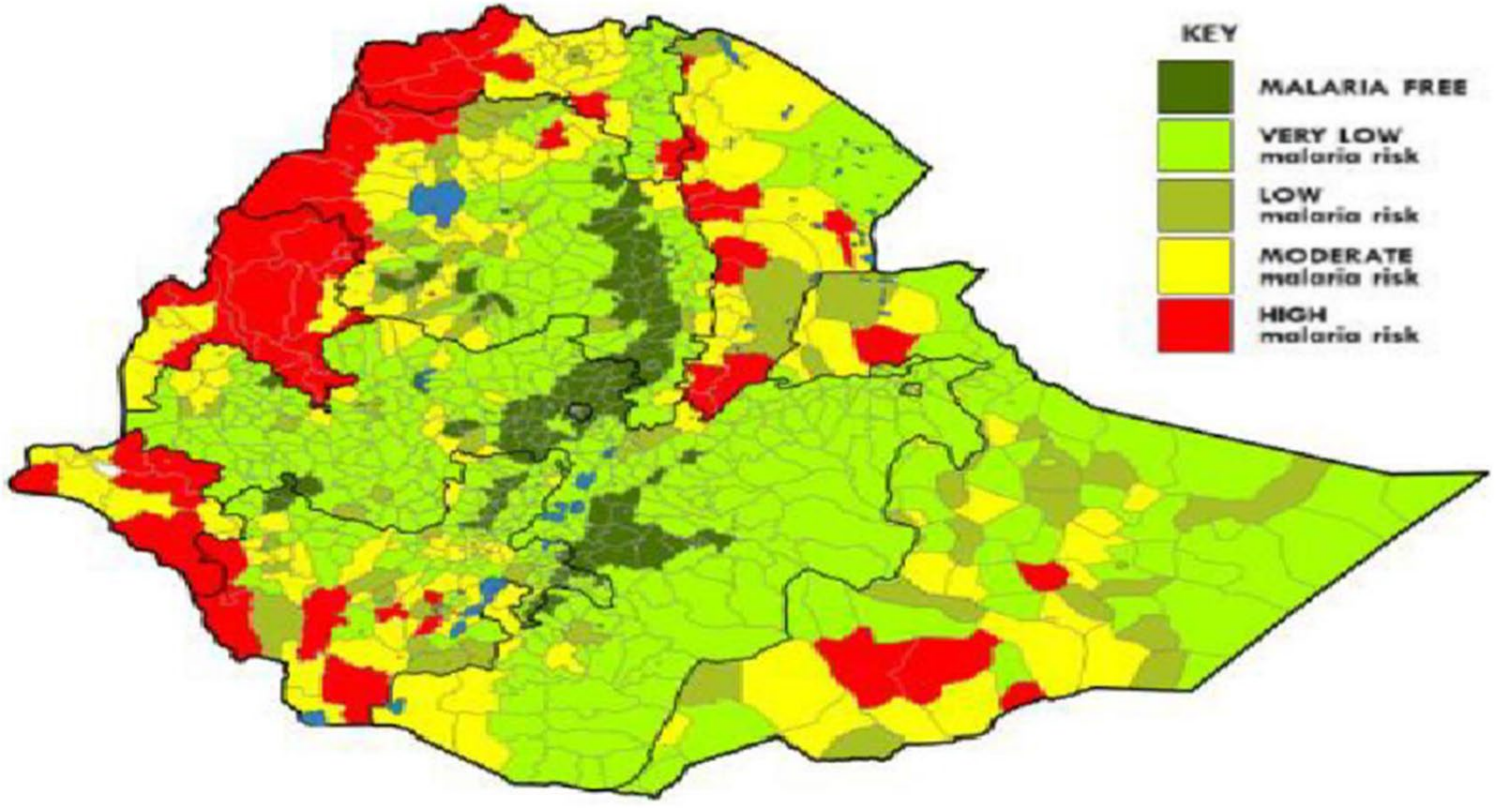
Ethiopia Malaria risk stratification, 2020 [39]

This study was carried out from September through December 2020 in two areas of western Ethiopia, Assosa and Anger Gute, with different levels of malaria transmission. From the high transmission area, the samples were collected from Sherkole and Horazhab health centres of Assosa zone in the Benishangul-Gumuz Region of Ethiopia. Sherkole health centre is located in the Sherkole district which is bordered by Sudan in the north. Sherkole town is about 754 km from Addis Ababa. Horazhab health centre is located in Kurmuk district, and it is bordered by Sudan in the north and west. It is about 769 km from Addis Ababa. Sherkole and Kurmuk districts border Eth-Sudan where there is human mobility between the two countries. In addition, there is a Sherkole Refugee camp that inhabitant more than 14,000 Sudan and South Sudan immigrants [36]. So, there is a chance of imported *P. falciparum* from Sudan and South Sudan that might affect the *pfcrt* and *pfmdr1* polymorphism in those study districts. Sherkole and Kurmuk districts are located in areas along the western borders with Sudan**,** characterized by high malaria transmission intensity [37, 38]. In Ethiopia, high malaria-risk areas are mainly located in the western lowland areas of the country [39] (Fig. 1).

**Fig. 2:**
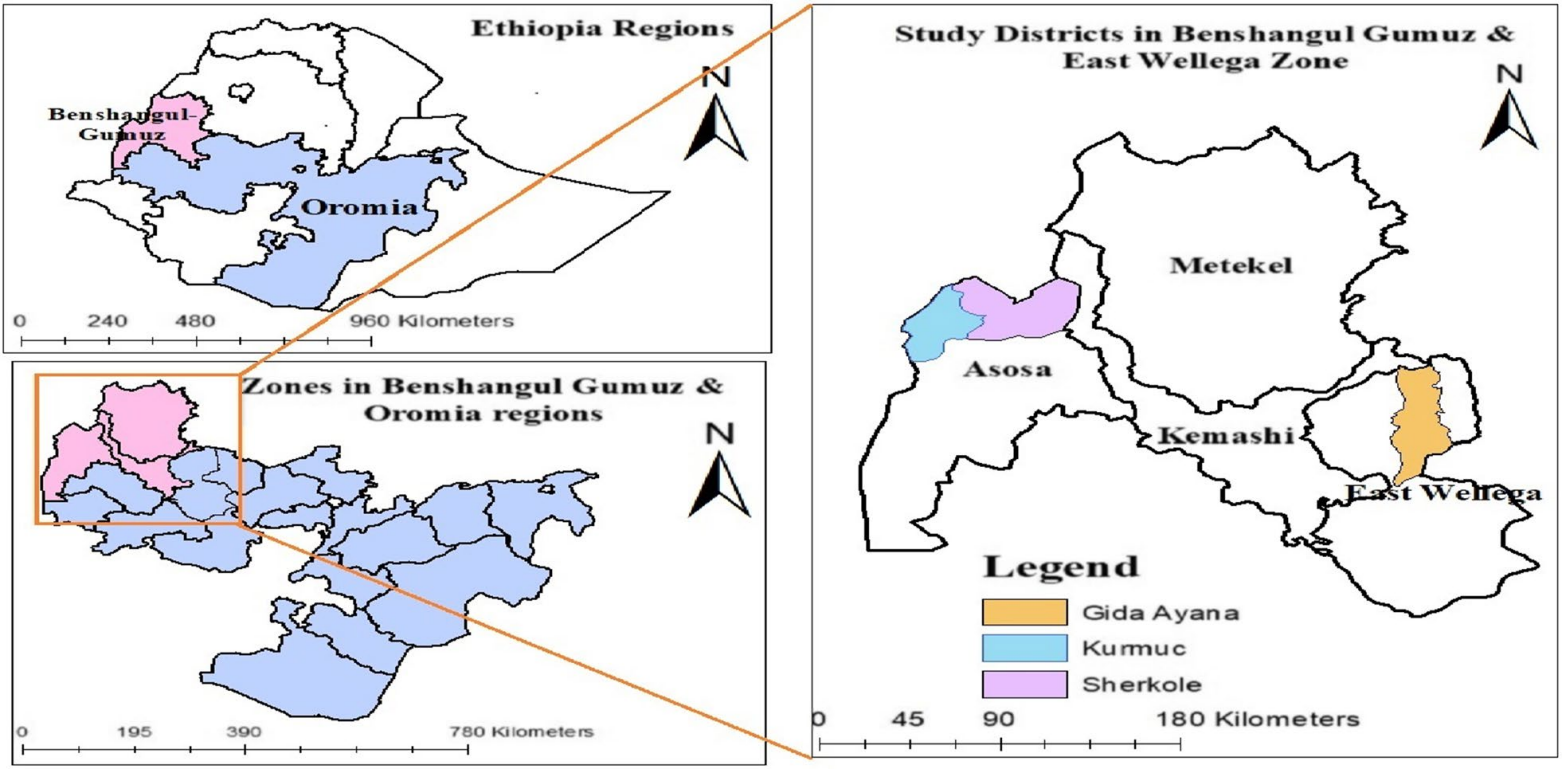
Study area map, Gida Ayana, Sherkole, and Kurmuk districts, Western Ethiopia

From the low transmission area, the samples were collected from Anger Gute and warabo health centers located in Gida Ayana district, East Wollega Zone, Oromia Regional State (Fig. 2). In the Anger Gute area malaria transmission is low and stable and *P. falciparum* infection prevalence among children 2–10 years was < 5% [37]. The incidence of malaria in and around Anger Gute town was 3.43 per 1000 population at risk of the disease, and the malaria trend from 2014 to 2018 indicated nearly unchanged numbers of malaria cases [40].

### Study design and population

A health facility-based cross-sectional study was conducted. At each study health center, patients with uncomplicated malaria whose age was greater than 6 months, and who were microscopically-confirmed to be infected with *P. falciparum***,** were enrolled.

### Sample size

The sample size was determined using a single population formula, a 13.1% prevalence of malaria in the Benishangul-Gumuz regional state [41], a 95% confidence level and a 5% precision. Accordingly, the calculated sample size was 175. With a 23% adjustment for the non-response rate, 216 uncomplicated falciparum malaria patients were included in the study. The numbers of patients included in the study were proportional to the number of confirmed uncomplicated falciparum malaria cases reported from the study health facilities in 2019. Accordingly, a total of 230 *P. falciparum* isolates, 112, 51, and 67, were collected from Sherkole, Kurmuk, and Gida Ayana districts, respectively.

### Microscopic diagnosis

For confirmatory diagnosis of *P. falciparum,* capillary blood samples were collected and used for preparation of thick and thin blood films for slide microscopy examination. Each slide was stained with 10% Giemsa for 10 min, and 100 fields were examined before designating a negative sample [42]. Once the patients were diagnosed for *P. falciparum* infection as determined by microscope, they gave finger-prick blood samples to prepare dried blood spots (DBS) on filter paper (Whatman No. 1001 320, International Ltd. Maidstone, England) for molecular analyses. The DBS were individually kept in plastic bags with desiccants until molecular biology analysis. Patients with mixed infections with other *Plasmodium* species were excluded from the study.

### Molecular genotyping

Parasite genomic DNA extraction was done from DBS using the Chelex protocol as earlier described [43]. NanoDrop™ Spectrophotometer did quantification of the DNA for extracted samples. All samples were normalized to 30 ng/µL DNA concentrations. Molecular genotyping was done at Medical Research Council Unit Gambia at the London School of Hygiene and Tropical Medicine.

*Plasmodium falciparum* detection was performed by *var* gene acidic terminal sequence (*varATS*) real-time PCR as previously described [44]. Parasites drug resistance genotyping assays were done using high-resolution melting assay (HRM) with a Light-Cycler^®^480 real-time polymerase chain reaction (PCR) machine and previously described procedure [45].We evaluated loci of *pfcrt* associated with chloroquine resistance, *Pfcrt* haplotype C72/M74/N75/K76 was considered wild-type and any polymorphism at any of these amino acids positions was considered mutant by HRM analysis.

*Pfmdr1* codon positions 86, 184, 1042 and 1246 were analysed for SNPs. In addition, the detection of *Pfmdr1* CNV was performed using a Bio-Rad CFX96 real-time PCR (Qiagen, Valencia, USA) as per the published protocol [46] and calculated using the formula 2^−ΔCt^ (ΔCt = Ct *PfMDR1*-Ctβ*-tubulin*). *PfMDR-1* copy number > 1.6 is defined as an amplification of the gene. All PCR-positive samples were examined by HRM across the two drug-resistance loci.

### Data analysis

The prevalence of *Pfcrt* wild or mutant parasites was determined by counting the number of samples observed with wild or mutant by HRM analysis divided by the total number of samples that were successfully genotyped by HRM for *Pfcrt* locus*.* Similarly*,* the prevalence of different SNPs in the *pfmdr1* gene was determined by counting the number of samples with a particular allele determined by HRM divided by the total number of samples that were successfully genotyped by HRM in a given codon.

Frequencies of *pfmdr1* haplotypes at codons N86Y, Y184F and D1246Y and *Pfmdr1*copy numbers were determined. Logistic regression analysis was done to show an association between the antimalarial drug resistance markers between the study sites. A *P*-value of less or equal to 0.05 was considered statistically significant.

### Ethical considerations

Ethical clearance was obtained from the Ethiopian National Ethics Review Committee and Addis Ababa University, Aklilu Lemma Institute of Pathobiology, IRB. The relevant regional and district health authorities sought permission to conduct the study at the health facilities. Written informed consent was obtained from adult study participants and a parent or guardian of a child. Written informed assent was also taken from children.

## Results

Out of 230 microscopically confirmed *P. falciparum* cases enrolled in the study, 225 of them tested positive by PCR. Of these PCR positive samples, 72.4% (163/225) and 27.6% (62/225) were collected from Asossa and Gida Ayana sites, respectively. The mean age of the study participants was 17.8 ± 12.7 years and with an age range of 7 months to 75 years old. The ratio of males to females was 1.27: 1.

HRM of *pfcrt* genes, including residues 72–76, was successfully for 95.5% (215/225) of the samples. Overall, the proportions of parasites carrying the wild-type CVMNK and the mutant haplotypes were 51.6% (111/215) and 46.5% (100/215), respectively. Mixed infections accounted for 1.9% (4/215). A total of 33.5% (52/155) and 80% (48/60) of isolates collected from the Assosa and Gida Ayana sites carry the mutant haplotypes, respectively suggesting that isolates circulating at Gida Ayana area carry the mutant genotypes as compared with the Assosa area (COR = 8.4, *P* = 0.00) (Table 1).

**Table 1:** *Pfcrt* haplotypes distribution and association with study sites in West Ethiopia

Genotype	Total	Study sites		COR	*P*-value
		Assosa (155)	Gida Ayana (55)		
Wild-type, n (%)	111 (51.6%)	100	11	1	
Mutant, n (%)	100 (46.5%)	52	48	8.4	0.00
Mixed, n (%)	4 (1.9%)	3	1	3.03	0.355

*COR* crude odds ratio

For the *pfmdr1* gene, 94.4%, 86.7%, 91.1% and 94.2% samples were successfully genotyped at codons 86, 184, 1042 and 1246, respectively. The N86Y wild type was detected among 79.8% (166/208) of the samples, of which 77% (114/148) were from Assosa and 86.7% (52/60) were from Gida Ayana. The Y184F mutation was found in 73.4% (146/199) samples, of which 105 were from Assosa and 41 were from Gida Ayana. None of the isolates carry N1042D mutation in this study. In addition, 89.6% (190/212) of parasites in west Ethiopia carry the wild-type D1246Y variants. There was no significant difference in *pfmdr1* SNPs between the two study sites (*P* > 0.05) (Table 2).

**Table 2:** *pfmdr1* SNPs distribution and association with study sites in western Ethiopia

Codon	Genotype	Total	Study areas		COR	*P*-value
			Assosa (163)	Gida Ayana (62)		
86 (n = 208, %)	N86	166 (79.8%)	114	52	1	
	86Y	33 (15.9%)	27	6	0.487	0.135
	Mixed	9 (4.3%)	7	2	0.63	0.57
184 (n = 199, %)	Y184	53 (26.6%)	40	13	1	
	184F	146 (73.4%)	105	41	1.2	0.62
1042 (n = 205, %)	N1042	205 (100%)	150	55		–
1246 (n = 212, %)	D1246	190 (89.6%)	135	55	1	
	1246Y	19 (10%)	15	4	0.65	0.47
	Mixed	3 (1.4%)	2	1	1.23	0.87

**Fig. 3:**
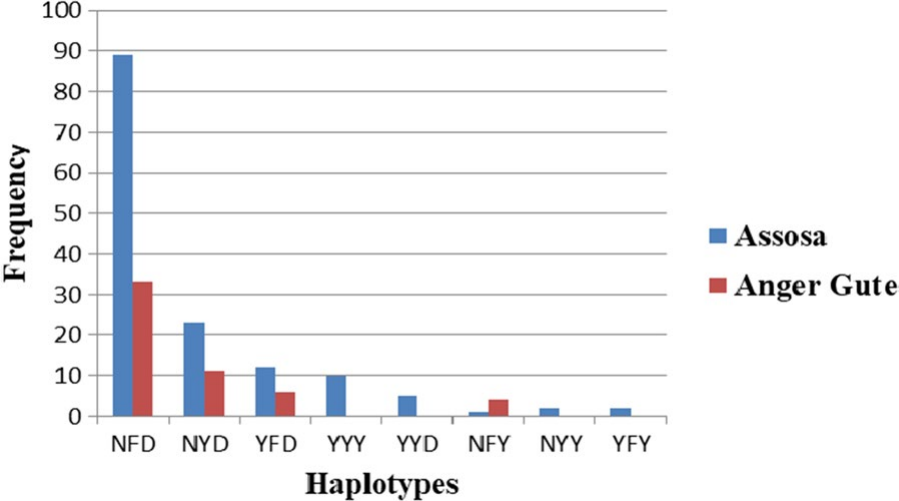
Distribution of *pfmdr1* haplotypes at N86Y–Y184F–D1246Y among *P. falciparum* isolates collected from two sites in west Ethiopia

*Pfmdr1* haplotypes were determined by combining SNPs at *pfmdr1* codons 86, 184 and 1246 and 89% (200/225) of samples were successfully genotyped at the three *pfmdr1* loci. Eight *pfmdr1* haplotypes at the N86Y–Y184F–D1246Y were identified among *P. falciparum* isolates in West Ethiopia. The NFD haplotype was observed in 61% (122/200) of the isolates and the NYD wild-type haplotype was found at a frequency of 17% (34/200) (Fig. 3).

While the haplotypes NFD, NFY, NYD and YFD were identified in both study areas; the YYD, YFY and YYY haplotypes were found only in clinical isolates collected from the Assosa site. More importantly, there was no statistically significant difference in the distribution of N86Y–Y184F–D1246Y haplotypes between the two study sites (*P* > 0.05) (Table 3).

**Table 3:** Distribution of *pfmdr1*haplotypes at N86Y–Y184F–D1246Y among *P. falciparum* isolates in West Ethiopia

Haplotypes	Study sites		COR	*P*-value
	Assosa	Gida Ayana		
NYD (n = 34)	23	11	1	
NFD (n = 122)	89	33	0.775	0.544
NFY (n = 5)	1	4	8.364	0.071
YFD (n = 18)	12	6	1.045	0.943

From a total of 225 samples that were successfully analysed for CNV in the *pfmdr1* gene, the proportion of parasites with multiple copies greater than 1.6 was 8.4% (19/225). The maximum copy number detected was 4.8. No statistically significant difference in *pfmdr1* CNV between the studies sites (*P* > 0.05) (Table 4).

**Table 4:** Distribution of *pfmdr1* gene copy number variation among *P. falciparum*isolates in West Ethiopia

Study sites	*Pfmdr1* CNV		COR	*P*-value
	Single	Multiply copy		
Assosa (n = 163)	149	14	1	0.89
Gida Ayana (n = 62)	57	5	0.934	

## Discussion

Surveillance of anti-malarial molecular markers is important to scan the persistence of known mutations and/or temporal genetic changes in the parasite population exposed to changing drug pressures. This study compares directional changes of *pfcrt* haplotype and *pfmdr1* genes polymorphism among parasites in the western part of Ethiopia that have been exposed to similar drug pressures but found in different levels of malaria transmission settings.

The wild-type *pfcrt* haplotype was detected among 51.6% (111/215) of *P. falciparum* isolates. This study showed the gradual return of the wild-type strains in west Ethiopia after more than two decades of removal of CQ pressure. This finding was in contrast to studies conducted in other parts of Ethiopia; South-central Oromia [13], East Shoa and West Arsi [14] and Southern Ethiopia [47] were all isolates were mutant and also with studies in East Shewa zone [15], Gonder [35] and North, south and east part of Ethiopia [48] that shown majority of parasites was mutant. The observed level of variation in a reversal of mutations associated with CQ resistance might be related to the dominance of *P. falciparum* infection in western Ethiopia compared with high levels of *Plasmodium vivax* co-endemicity in the country, reflecting the discrepancy of drug pressure in different geographical areas of the country [16].

The proportion of *P. falciparum* isolates with mutant *pfcrt* haplotype was higher in the Gida Ayana area compared with Assosa. This variation in the study sites might relate to the difference in the level of malaria transmission between the two sites. The low malaria transmission in the Gida Ayana area might fix the chloroquine-resistant *P. falciparum* as compared to the high malaria transmission site, Assosa. Spread of *P. falciparum* resistance malaria has been associated with intensity of malaria transmission [49, 50]. Thus, after withdrawal of chloroquine, the spread of chlorquine sensitive parasites is higher in Asossa where malaria transmission is high compared with Gida Ayana in which transmission is low. On the other hand, the difference might also associate with the different levels of *P. vivax* co-endemicity between the two study areas and the continued availability of CQ for treating *P. vivax* [16]. In 2019/2020, the prevalence of *P. vivax* infection in the study districts was 24.6% (167/680) in the Gida Ayana district [51] compared with 0.1% (74/12,358) and 1.9% (72/3860) in Sherkole and Kurmuk districts of Assosa, respectively [52]. Therefore, the fixation of the CQ-resistant haplotype in the Anger Gute area was likely related to CQ drug pressure used to treat *P. vivax* infections.

The wild-type *Pfmdr1*-N86 allele that related to reduce sensitivity to lumefantrine was observed in 79.8% (166/208) of the investigated *P. falciparum* clinical isolates. This prevalence was in agreement with a past study reported from the study site, Benishangul-Gumuz [16]. Our finding was also consistent with other studies conducted in other parts of Ethiopia; Southeast Ethiopia [7], Adama and Gambella [14]. This study also showed that 73.4% (146/199) of the parasites carried the 184F variant that related to reduced susceptibility to mefloquine and lumefantrine. This finding was comparable to previous reports from North, South and east Ethiopia (48) and Southwest Ethiopia (26). Thus, a successive use of AL treatment has induced the selection of the *pfmdr1*-N86 and *pfmdr1*-184F strains that reduce parasite susceptibility to lumefantrine in the study area.

At codon N1042D, no mutation was observed in clinical isolates collected from west Ethiopia. The absence of mutation at this codon was previously reported from Southeast Iran [53], Saudi Araba [22] and Vanuatu and Solomon Islands [54]. However, the finding was in contrast to studies from South America that reported mutation of the N1042D [20, 55], and this SNP contributes to resistance to quinine [56]. Therefore, distribution of *pf*mdr1-N1042D mutation might differ over different geographic areas due to the discrepancy in the genetic variation of the parasites and the type of stress that each strain had experienced. It also demonstrates the low or no contribution of the N1042D mutation to antimalarial drug pressure in the study area.

The majority (89.6%) of parasites in west Ethiopia carried the wild-type D1246 allele. The observed high level of the wild-type D1246 allele was in line with previous studies conducted in Southeast Ethiopia [27], NorthernUganda [57], and East-Central Gabon [58]. Therefore, the high frequency of D1246 was likely associated with selective pressures of AL treatment in the study area as parasites with genotypes N86, 184F, and D1246 are partially resistant to the treatment [23].

This study identified the presence of eight *pfmdr1*haplotypes at the N86Y–Y184F–D1246Y circulating in west Ethiopia, with the NFD haplotype dominant (61%) followed by the wild type NYD (13.7%). The dominance of the NFD haplotype in this study was in agreement with previous studies in Africa [9, 23, 59]. Thus, a widespread use of AL as first-line anti-malarial treatment raises the NFD haplotype selection that reduces parasite sensitivity to lumefantrine. Although AL remained efficacious in the study area, persistence of residual submicroscopic *P. falciparum* parasitaemia following the treatment need continuing surveillance using genetic markers may help track the spread of lumefantrine-resistant parasites [60].

In the present study, 8.4% of isolates harbor multiple copies of *pfmdr1*. This prevalence was comparable to studies done in Kenya [30], Ghana [59], Mynamar [32] and Suriname [33]. However, it was lower than the amplification of the gene reported in North part of Ethiopia [35], Venezuela [34] and Thailand [61]. The difference might be related to underlying genetic background of the parasites and drug pressure caused by antimalarial drugs used in the study sites.

There were no significant differences in the distributions of *pfmdr1* SNPs at codons 86, 184, 1042 and 1246. Similarly, *pfmdr1* haplotypes and CNV were not associated with study sites (*P* > 0.05). Thus, frequencies of the mutations are not related to geographic selection and level of malaria transmission but are associated with rates of selection to AL treatment [23].

## Conclusion

There is a gradual regaining of chloroquine-sensitive haplotype, and the return is more in areas of high transmission than low transmission sites in western Ethiopia with principally *P. falciparum* infections. A high prevalence of the wild-type alleles N86, D1042 and D1246 and of the mutant-type allele 184F was detected in the study sites. The NFD haplotype was the predominant haplotype of the N86Y–Y184F–D1246Y, and 8.4% of the parasite carried multiple copies of the *pfmdr1* gene. Continuous investigation is needed to closely monitor the changes in the *pfmdr1* SNPs associated with the selection of *P. falciparum* parasites populations by ACT.

## Data Availability

The datasets and analysed results of the study are available from the corresponding author and can be obtained on reasonable request.

## Abbreviations

ACT: Artemisinin-based combination therapy
AL: Artemether–lumefantrine
CQ: Chloroquine
*Pfcrt*: *P. falciparum* chloroquine-resistant transporter
CNV: Copy number variations
DBS: Dried blood spots
HRM: High-resolution melting assay
IRB: Institute Review Board
PCR: Polymerase chain reaction
*pfmdr1*: *P. falciparum* multidrug resistance 1
*VarATS*: Var gene acidic terminal sequence
SNP: Single nucleotide polymorphisms
